# Supplementary material for: Transcriptomic Analysis Identifies Molecular Response of the Tolerant Alfalfa (Medicago sativa) Cultivar Nongjing 1 to Saline-Alkali Stress
Source: Biology (Basel). 2025 Apr 18;14(4):439. doi: 10.3390/biology14040439 (PMC12024754; doi:10.3390/biology14040439)
Supplement: Supplementary file 1 [file biology-14-00439-s001.zip › Supplementary figures 4.15.pptx]

## Slide 1
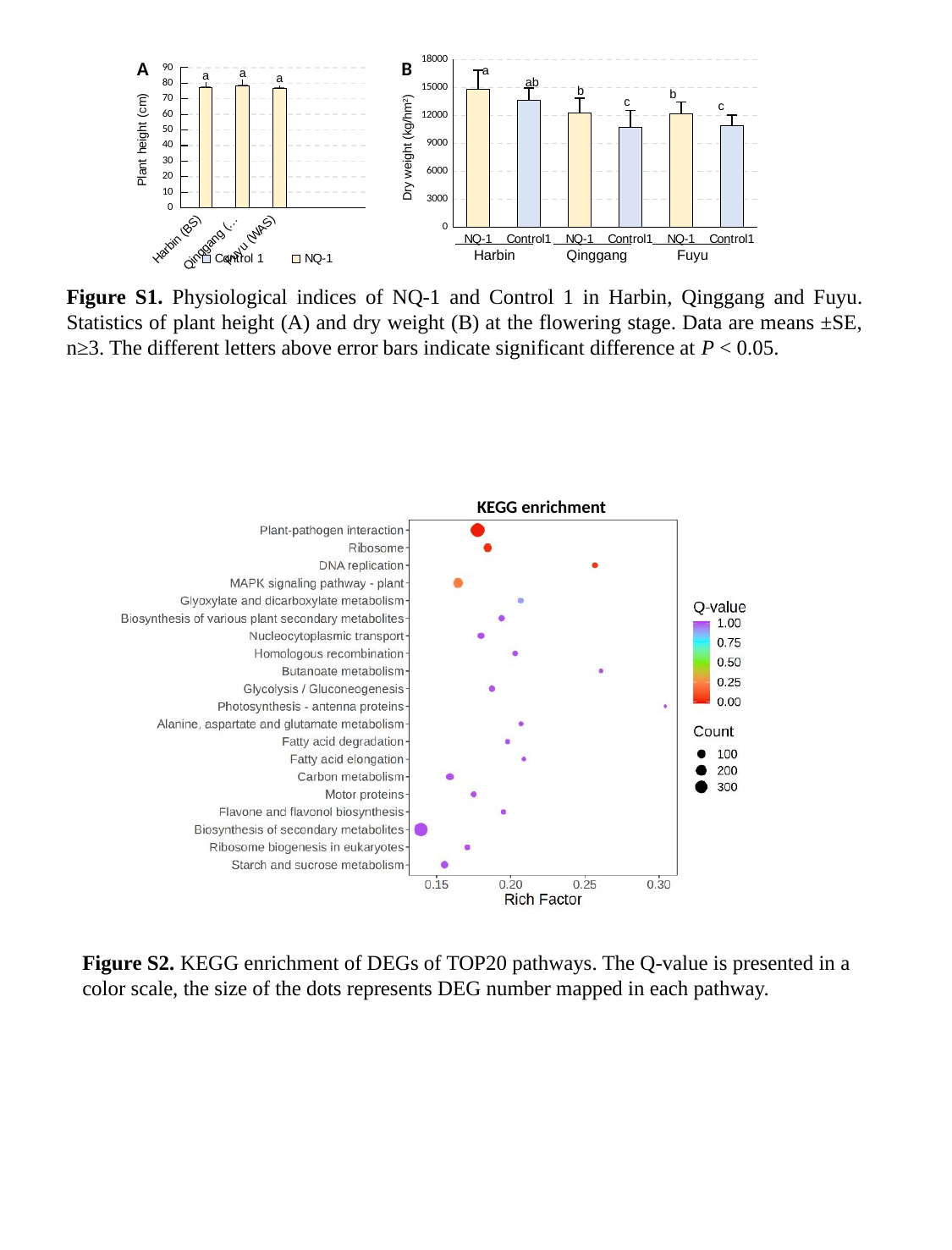

B
### Chart
| Category | |
|---|---|
| NQ-1 | 14783.3333333333 |
| Control1 | 13616.6666666667 |
| NQ-1 | 12250.0 |
| Control1 | 10750.0 |
| NQ-1 | 12133.3333333333 |
| Control1 | 10900.0 |Dry weight (kg/hm2)
Harbin
Qinggang
Fuyu
a
ab
b
b
c
c
A
### Chart
| Category | Control 1 | NQ-1 |
|---|---|---|
| Harbin (BS) | 76.5333333333333 | 77.0666666666667 |
| Qinggang (SCL) | 72.6 | 78.0 |
| Fuyu (WAS) | 71.3333333333333 | 76.4 |Figure S1. Physiological indices of NQ-1 and Control 1 in Harbin, Qinggang and Fuyu. Statistics of plant height (A) and dry weight (B) at the flowering stage. Data are means ±SE, n≥3. The different letters above error bars indicate significant difference at P < 0.05.
KEGG enrichment
Figure S2. KEGG enrichment of DEGs of TOP20 pathways. The Q-value is presented in a color scale, the size of the dots represents DEG number mapped in each pathway.

## Slide 2
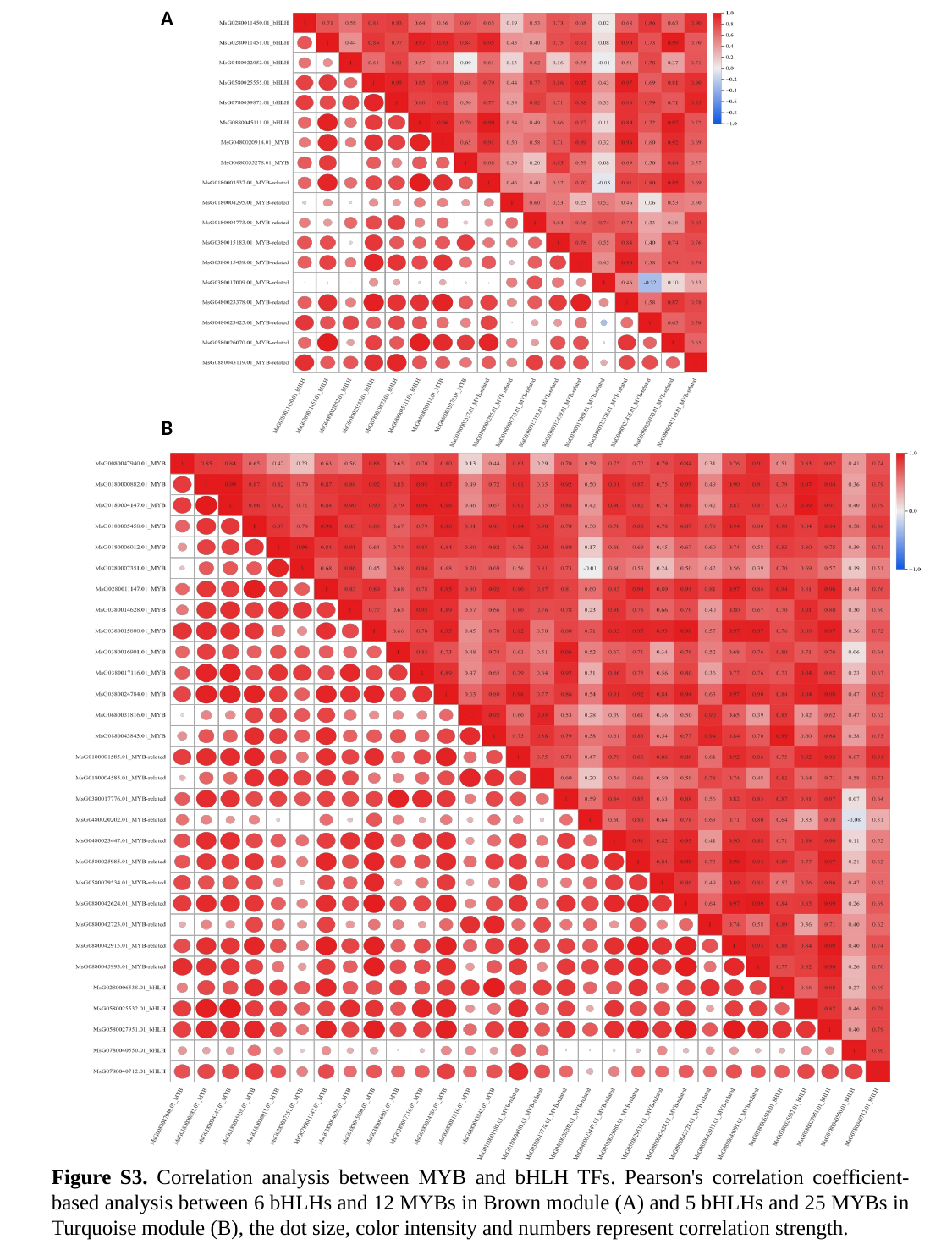

A
B
Figure S3. Correlation analysis between MYB and bHLH TFs. Pearson's correlation coefficient-based analysis between 6 bHLHs and 12 MYBs in Brown module (A) and 5 bHLHs and 25 MYBs in Turquoise module (B), the dot size, color intensity and numbers represent correlation strength.
