# Supplementary material for: Transcriptomic Analysis Identifies Molecular Response of the Tolerant Alfalfa (Medicago sativa) Cultivar Nongjing 1 to Saline-Alkali Stress
Source: Biology (Basel). 2025 Apr 18;14(4):439. doi: 10.3390/biology14040439 (PMC12024754; doi:10.3390/biology14040439)
Supplement: Supplementary file 1 [file biology-14-00439-s001.zip › Table S1 Statistics of information on turning green 4.15.pdf]

Table S1 Statistics of information on turning green (2003 and 2004).

Location	Variety / Cultivar	Turn green date (mm/dd)	Average rate of turning green (%)
Harbin	NQ-1	4/7	99.8
	C1	4/7	99.5
Qinggang	NQ-1	4/22	98
	C1	4/25	97
Fuyu	NQ-1	4/12	98.1
	C1	4/14	98
